# Targeting the Hippo/YAP/TAZ signalling pathway: Novel opportunities for therapeutic interventions into skin cancers

**DOI:** 10.1111/exd.14655

**Published:** 2022-08-12

**Authors:** Alexander Howard, Jodie Bojko, Benjamin Flynn, Sophie Bowen, Ute Jungwirth, Gernot Walko

**Affiliations:** ^1^ Department of Life Sciences University of Bath Bath UK; ^2^ Centre for Therapeutic Innovation University of Bath Bath UK

**Keywords:** basal cell carcinoma, epidermis, Hippo signalling, melanoma, skin cancer, squamous cell carcinoma

## Abstract

Skin cancers are by far the most frequently diagnosed human cancers. The closely related transcriptional co‐regulator proteins YAP and TAZ (WWTR1) have emerged as important drivers of tumour initiation, progression and metastasis in melanoma and non‐melanoma skin cancers. YAP/TAZ serve as an essential signalling hub by integrating signals from multiple upstream pathways. In this review, we summarize the roles of YAP/TAZ in skin physiology and tumorigenesis and discuss recent efforts of therapeutic interventions that target YAP/TAZ in in both preclinical and clinical settings, as well as their prospects for use as skin cancer treatments.

## 1 INTRODUCTION

Skin is the largest organ of the human body and has important barrier, sensory and immune functions.^1^, ^2^ In the skin, various cell populations cooperate to provide protection from daily wear and tear, harmful microorganisms and other attacks from the external environment. In addition, skin enables thermoregulation and tactile sensations.^1^, ^2^

Skin cancers are by far the most frequently diagnosed human cancers.^3^, ^4^, ^5^, ^6^ While non‐melanoma skin cancers are more common, melanoma is the most dangerous type due to its ability to metastasize.^5^, ^7^

In the skin, the Hippo signalling pathway and its downstream effectors, the transcriptional co‐regulator proteins Yes‐associated protein (YAP) and transcriptional co‐activator with PDZ‐binding motif (TAZ, also called WW Domain Containing Transcription Regulator 1 (WWTR1)), regulate diverse tissue‐specific functions during development, homeostasis and regeneration.^8^ The Hippo pathway is a tumor suppressor pathway, since its deregulation and the resulting YAP/TAZ hyperactivation promote development and progression of many cancer types, including skin cancers.^8^, ^9^, ^10^, ^11^ Importantly, there is strong evidence that YAP/TAZ are essential in both melanoma and non‐melanoma skin cancers,^8^, ^10^ but they appear to be largely dispensable for normal tissue homeostasis,^12^, ^13^, ^14^ pinpointing YAP/TAZ as interesting novel therapeutic targets.

We begin this review by giving a brief overview of the contributions of different skin cell populations to tissue homeostasis and repair and the roles of these cells in cancer development. In the second part, we summarize the specific roles of Hippo/YAP/TAZ signalling in controlling cell functions in healthy skin, review the roles of Hippo/YAP/TAZ signalling in skin cancer development and progression and discuss potential therapeutic approaches. We close this review with discussing major open questions.

## 2 EPIDERMAL TISSUE HOMEOSTASIS AND REPAIR

**FIGURE 1:**
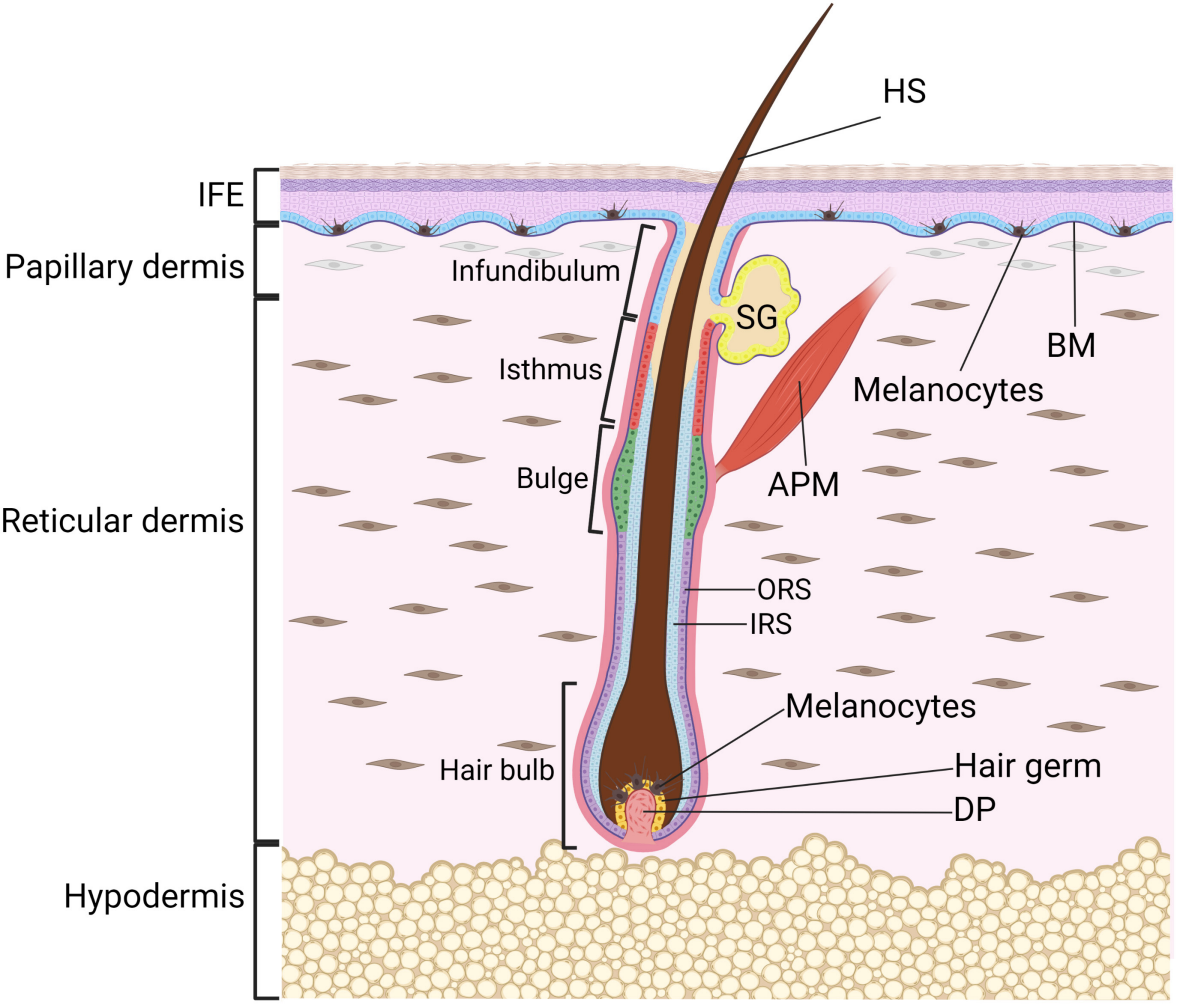
Morphology of the skin. The epidermis and the underlying dermis are separated by a basement membrane (BM). Multiple, spatially distinct stem cell populations have been identified in the interfollicular epidermis (IFE) and the bulge, isthmus, infundibulum and sebaceous gland (SG) parts of the hair follicle and are indicated by different colours. Two populations of fibroblasts populate the dermis: papillary fibroblasts are in proximity to the BM, while reticular fibroblasts are found in the central dermis. The hair follicle is depicted in the growth phase (anagen), when a transient population of stem cells in the hair germ create an inner root sheath (IRS) and hair shaft (HS, protruding out of the skin surface), while stem cells in the permanent bulge region of the hair follicle give rise to the outer root sheath (ORS). The hair germ rests above the dermal papilla (DP), a population of mesenchymal cells that provides inductive signalling for hair growth and modulates hair follicle regeneration. Pigment‐producing melanocytes are present in the hair follicle and the IFE. APM, arrector pili muscle. Figure graphics were created with BioRender.com.

Skin epidermis comprises the interfollicular epidermis (IFE) and associated epidermal appendages (Figure 1). These include pilosebaceous units, consisting of a hair follicle (HF) and sebaceous and sweat glands.^2^, ^15^, ^16^ In addition to keratinocytes, mammalian epidermis contains several other cell types, including melanocytes, Merkel cells, gamma delta (γδ) T‐cells and Langerhans cells^1^ (Figure 1). The IFE consists of several layers of suprabasal keratinocytes at various stages of a terminal differentiation programme, and a basal layer of proliferative keratinocytes which express keratins KRT5 and KRT14 and are attached to the underlying basement membrane (BM) via integrin (ITG) extracellular matrix receptors.^1^, ^2^, ^8^

Continuous renewal of the IFE and its appendages throughout life is ensured by stem cells (SCs) and progenitor cells that balance proliferation and differentiation to replace dead and terminally differentiated cells.^1^, ^2^, ^8^, ^15^, ^16^ During tissue homeostasis, HFs undergo continuous cycles of growth (anagen) and degeneration (catagen), followed by a resting stage (telogen).^1^ The SCs responsible for cyclic HF regeneration are located in the permanent non‐cyclic HF portion called the bulge^1^, ^15^, ^16^ (Figure 1). The upper portion of the HF does not cycle but turns over frequently, which is governed by multiple resident SC pools^15^, ^16^, ^17^ (Figure 1). The identity, organization and dynamics of SCs within the IFE are still matters of debate.^18^ Current models of mouse IFE homeostasis suggest that each basal cell appears to be equipotent and generates progeny that have equal probability to self‐renew or differentiate.^18^ In contrast, the human IFE appears to be maintained by a hierarchy of SCs that generates actively dividing progenitor cells which ultimately commit to terminal differentiation.^18^, ^19^, ^20^

In wounded mouse skin, several epidermal cell populations, including those in HFs distal to the wound site, contribute to the skin wound repair process.^15^, ^16^ Interestingly, lineage restriction and spatial confinement of HF‐resident SC pools are transiently lost during wound repair, allowing contribution of multiple SC populations.^1^, ^15^, ^16^ This lineage plasticity is not only critical in wound repair but is also functional in skin cancer development.^16^, ^21^

## 3 SKIN CANCER

Skin cancers are among the most frequently diagnosed human malignancies world‐wide, with over a million cases detected each year.^4^, ^22^ Skin cancers can be divided into cutaneous melanomas (CM, also referred to as malignant melanoma of the skin, or melanoma skin cancer) and non‐melanoma skin cancers (NMSC).^4^, ^6^, ^22^ NMSCs comprise several different types of carcinomas, such as basal cell carcinomas (BCC), cutaneous squamous cell carcinomas (cSCC), keratoacanthomas, Merkel cell carcinomas and various rare adnexal tumors, as well as angiosarcomas and cutaneous lymphomas.^3^, ^4^, ^6^, ^8^ Approximately 80% of NMSCs are BCC, and 20% are cSCC.^3^, ^4^ These two most common types of NMSC, originating from epidermal keratinocytes, are nowadays also referred to as keratinocyte cancers.^3^, ^23^

### 3.1 Non‐melanoma skin cancer

Non‐melanoma skin cancers incidence greatly outnumbers CM, but most NSMCs are much easier to treat and have much better long‐term prognosis, especially when detected at their initial stages.^6^ NMSC most frequently occur on commonly sun‐exposed parts of the body, and affect mostly persons with fair skin, who tend to burn easily rather than suntan when exposed to sunlight.^3^, ^4^ The long‐term, repeated exposure to ultraviolet (UV) radiation, both solar and artificial, is a causative factor for nearly 90% of NMSCs.^3^, ^4^, ^6^ Yet, despite increasing public awareness of the harmful effects of sun exposure and health costs, NMSC incidence has been increasing by 4% each year.^3^, ^22^

Basal cell carcinoma is a malignant cancer that arises from the basal epidermal cell layer.^4^, ^24^ BCC is generally only locally invasive and rarely metastasizes.^3^, ^4^ BCCs do not proliferate rapidly, but, if ignored and left untreated, they are prone to destroy local underlying tissues.^3^ Chronic exposure to UV radiation plays the most important role in BCC pathogenesis.^3^, ^4^ However, individual risk factors for BCC also include gender, age and genetic diseases (e.g. Gorlin–Goltz syndrome),^3^, ^6^, ^24^ exposure to ionizing radiation, carcinogenic chemicals (especially arsenic) and immune suppressive drugs.^4^ Deregulation of the hedgehog(HH)/PTCH1/SMO signalling pathway is central to BCC development.^24^ Hyperactivation of the HH pathway, either through deletion of *PTCH1*, mutational activation of SMO or overexpression of *GLI1* or *GLI2*, has been reported in human and mouse BCC.^3^, ^4^, ^24^ BCCs display different growth patterns and can be classified into various subtypes based on tumor location, gender, age and skin type.^3^, ^24^ A number of lineage tracing studies (summarized in^24^) that used Cre‐mediated cell‐specific targeting in mice, either by lineage tracing or by the activation of oncogenic HH signalling in distinct cell populations, have provided strong support for a stem/progenitor cell origin of BCC. The results from these studies also suggest that oncogenic HH signalling can drive BCC initiation in several different epithelial stem‐ and progenitor cell populations in mouse skin, including SCs in the bulge and isthmus regions of the HF and GLI1‐positive cells in touch dome epithelia, although the tumor morphology and the final outcome of BCC development are also influenced by the mutated HH signalling pathway member, and the strength of oncogenic HH signalling.^24^, ^25^, ^26^ In the context of human skin, experimental evidence suggests that BCCs originate from CD200‐positive SCs in the HF bulge.^27^

While BCC contributes minimally to the keratinocyte cancer mortality rate, cSCC accounts for about 75% of keratinocyte cancer‐related deaths.^3^, ^4^, ^23^ Like BCC, cSCC also arises from basal epidermal cells. It is characterized by infiltrative and metastatic behaviour as well as destructive growth.^3^, ^28^ Once cSCC has progressed to an invasive stage, it has the potential to recur after surgical removal and to metastasize, with a variable metastatic rate of 0.1%–9.9%, and with transplant recipients and immunocompromised patients being at greater risk of developing metastatic disease.^3^, ^4^, ^29^ The mortality rate of patients with distant metastatic cSCC is very high (70%–89%), and a curative therapeutic approach is still lacking.^3^, ^29^ Similar to BCC, solar UV radiation is an important risk factor for the development of cSCC and leads to genetic and epigenetic changes both in basal epidermal cells and cells of the underlying dermal stroma.^23^, ^28^, ^30^ Consequently, cSCC most commonly arises in sun‐damaged skin. One of the strongest predictors of cSCC development in previously unaffected people is the presence of actinic keratoses (AKs, also known as solar keratoses or cSCC in situ), which are benign scaly dysplastic keratinocyte‐derived tumors caused by cumulative sun exposure.^23^, ^28^ Other risk factors include infection with human papillomaviruses and genetically inherited cutaneous diseases, such as albinism, xeroderma pigmentosum and epidermodysplasia verruciformis.^4^, ^23^ However, there are also clinical situations in which increased cSCC formation in patients is associated with therapeutic treatments.^28^ A noticeable example is cSCCs induced by long‐term treatment with immunosuppressive drugs.^31^ cSCC development is a complex process, but it is frequently associated with mutations in RAS GTPases (*HRAS* and *KRAS*), cell cycle regulators such as *TP53* and *CDKN2A*, regulators of squamous cell differentiation such as Notch signalling receptors (*NOTCH1*, *NOTCH2* and *NOTCH3*), chromatin remodelling factors such *KMTC2* and *KMTD2,* and *FAT1* cadherin.^4^, ^28^, ^30^ Dysregulated RAS/receptor tyrosine kinase/PI3K and cell cycle pathways appear to be particularly involved in aggressive cSCC.^32^ In mice, cSCC can be induced through multistage carcinogenesis models that use chemical carcinogens, UV irradiation or forced expression of oncogenes targeted to epidermal SC populations.^28^, ^30^, ^33^, ^34^ Using transgenic mice where the expression of oncogenic KRAS was targeted to different compartments of adult epidermis, studies found that both IFE and HF SCs are cells of origin of mouse cSCC.^30^ However, targeting of oncogenic KRAS to HF SCs led to formation of more aggressive cSCCs with features of epithelial to mesenchymal transition (EMT).^30^ Of note, a hybrid EMT state seems to be associated with cSCC metastasis.^35^

### 3.2 Cutaneous melanoma

Melanoma skin cancer is a malignancy of melanocytes. The cutaneous form of melanoma (CM) causes the majority (75%) of deaths related to skin cancers.^22^, ^36^, ^37^ CM is characterized by an extensive degree of heterogeneity in terms of clinical, dermatological and histopathological presentation,^36^, ^37^, ^38^ genomic profile,^37^, ^38^, ^39^ and risk factors.^36^, ^37^, ^38^, ^39^ The development of fully evolved CM from pre‐neoplastic lesions is complex and not represented by a single evolutionary pattern.^37^, ^38^, ^39^ The spectrum of melanocytic neoplasms ranges from benign nevi (circumscribed proliferations of melanocytes), which are common and have only a very marginal risk of progressing, to invasive melanomas, which have the potential to metastasize.^37^, ^38^ In between are several intermediate stages that include dysplastic naevi and non‐invasive (in situ) CM.^37^, ^38^ Sun (UV) exposure is the main environmental risk factor for CM development, and CM occurs mainly in white populations with fair skin.^36^, ^39^ According to the 2018 “WHO Classification of Skin Tumors,” melanomas can be divided into thosethat are causally related to sun exposure and those that are not, as determined by their mutational signatures, anatomic site and epidemiology.^38^ Based on their origins from skin that is or is not chronically sun damaged (CSD), CMs can be broadly categorized into high‐CSD, low‐CSD and non‐CSD.^37^, ^38^ High‐CSD CMs encompass lentigo maligna and desmoplastic melanomas, and low‐CSD CMs include superficial spreading melanomas.^38^ The non‐CSD (or variable/incidental UV radiation exposure) category includes acral melanomas, some melanomas in congenital nevi, melanomas in blue nevi, Spitz melanomas, but also non‐cutaneous melanomas, such as mucosal melanomas and uveal melanomas.^38^ High‐CSD CMs commonly arise from in situ CM on skin of older (>55 years of age) individuals with a history of long‐term exposure to UV radiation, and have a high mutational burden associated with *NF1*, *NRAS*, *BRAF* ^nonV600E^ or *KIT* driver mutations, leading to aberrant activation of the MAPK pathway.^37^, ^39^ In contrast, low‐CSD CMs most commonly arise from benign or dysplastic naevi, affect the more sporadically sun‐exposed areas of younger individuals (<55 years of age), and are often associated with a moderate mutational burden and predominance of *BRAF* ^V600E^ driver mutations.^37^, ^38^ In the absence of other driver mutations, the *BRAF* ^V600E^ mutation causes limited melanocyte proliferation which is kept in check by oncogene‐induced cell senescence.^37^ The resulting nevus remains stable for decades, also due to immune surveillance.^37^ In fact, melanomas are among the most immunogenic tumors.^40^ Progression of low‐ and high‐CSD CMs to invasive CM is usually associated with secondary and tertiary mutations, such as *TP53* and *PTEN* mutations, telomerase reverse transcriptase *(TERT)* promoter mutations and bi‐allelic loss of *CDKN2A*.^37^, ^39^ No conclusive hierarchic mutation pattern associated with metastasis has been identified, suggesting that metastatic progression involves distinct transcriptional programmes.^37^, ^41^ In the non‐CSD category of CM, Spitz melanomas are characterized by driver fusion genes including the kinase domains of *ALK*, *ROS1*, *NTRK1*, *NTRK3*, *MET*, *RET*, *BRAF* and *MAP3K8*, while Acral melanomas display a high incidence of copynumber variation with gene amplifications of *CCND1* and *KIT*.^38^

## 4 THE HIPPO SIGNALLING PATHWAY

**FIGURE 2:**
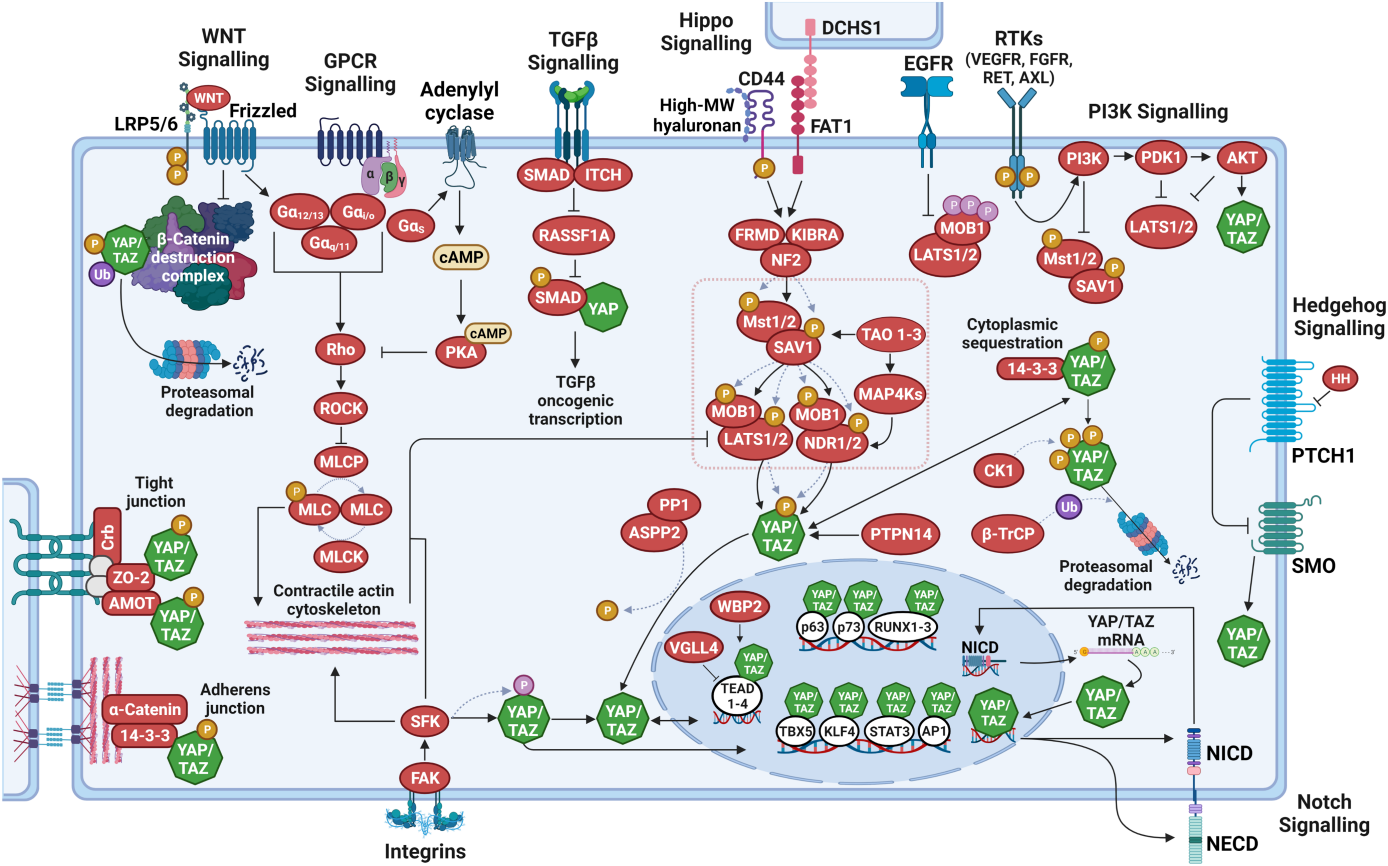
Key signals regulating YAP/TAZ activity. The transcription co‐regulators Yes‐associated protein (YAP) and transcriptional co‐activator with PDZ‐binding motif (TAZ), are predominantly regulated by phosphorylation (serine phosphorylation, orange; tyrosine phosphorylation, pink). Serine‐phosphorylated YAP/TAZ are exported from the nucleus and are either degraded in the cytoplasm via the proteasome or sequestered in the cytoplasm via 14‐3‐3 proteins or at tight‐ and adherens junctions. In their non‐serine‐phosphorylated and tyrosine‐phosphorylated states, YAP/TAZ accumulate in the nucleus, where they bind to various transcription factors, most notably those of the TEA domain (TEAD) family, to control target gene expression. In the nucleus, vestigial‐like family member 4 (VGLL4) competes with YAP/TAZ in binding to TEADs, while WW domain binding protein‐2 (WBP2) enhances the co‐activator functions of YAP/TAZ. The core of the Hippo pathway (dotted pink box) is defined by a kinase cascade composed of MST1 and MST2 kinases, large tumour suppressor (LATS)1 and LATS2 kinases and their co‐factors SAV1 and MOB1A and MOB1B. Membrane‐associated signalling events causing Hippo pathway activation include high‐molecular‐weight hyaluronan‐mediated clustering of CD44 and cell–cell signalling via Dachsous cadherin‐related 1 (DCHS1)/FAT1. Hippo pathway activation involves the phosphorylation of the core Hippo kinases, MST1/2 and LATS1/2: MST1/2 are autophosphorylated and subsequently phosphorylate LATS1/2. MST1/2 are also activated by TAO kinases. Activation of LATS1/2 causes the serine‐phosphorylation of YAP and TAZ and inhibits their transcription co‐regulator functions. PP1, together with apoptosis‐stimulating protein of p53 2 (ASPP2), antagonizes Hippo pathway activity by de‐phosphorylating YAP/TAZ. In addition to MST1/2, various upstream effectors of the LATS1/2 have been identified, including the MAP4K and TAOK families of kinases, which phosphorylate and activate LATS1/2. Nuclear Dbf2‐related (NDR)1/2 kinases act in parallel to LATS1/2 in the Hippo pathway to inactivate YAP/TAZ. The activities of the core Hippo pathway components are regulated by several upstream mechanisms. These involve various scaffolding proteins such as angiomotin (AMOT), neurofibromin 2 (NF2; also known as Merlin), kidney and brain protein (KIBRA; also known as WWC1), the protocadherin FAT1 and zonula occludens (ZO) proteins at tight junctions. Cell polarity and adhesion regulators promote LATS1/2‐mediated regulation of YAP/TAZ by altering actin dynamics and by facilitating Hippo pathway effector association. G protein‐coupled receptors (GPCR) signalling, mechanical cues and signals transduced by the extracellular matrix and matrix‐binding integrins (through FAK and SRC family kinases (SFKs)) can inactivate LATS1/2 by promoting a contractile F‐actin‐myosin cytoskeleton. SFKs also directly regulate YAP/TAZ nuclear abundance, predominantly by controlling their nuclear export rate. Soluble growth factors bind to and activate receptor tyrosine kinases (RTKs) and inactivate the Hippo pathway by stimulating PI3K–PDK1 signalling. EGFR activation causes inhibitory tyrosine phosphorylation of MOB1A/B. RASSF1A is recruited to the activated TGF‐b receptor I and subsequently targeted for degradation by the co‐recruited E3ubiquitin ligase ITCH. RASSF1A degradation then permits YAP association with SMADs and subsequent nuclear translocation of receptor‐activated SMAD2. YAP/TAZ are also regulated by WNT signalling: such as β‐catenin, YAP/TAZ also incorporates into the destruction complex and are targeted for proteasomal degradation. Upon WNT stimulation, inactivation of the destruction complex then drives β‐catenin as well as YAP/TAZ nuclear translocation. YAP/TAZ also interact with the Notch pathway: in the nucleus, YAP/TAZ can induce the gene expression of Notch receptors and/or Notch ligands to regulate Notch signalling, while the transcriptionally active Notch intracellular domain (NICD) can activate YAP1 gene transcription. Activated HH signalling leads to increased nuclear YAP abundance. AKT, Ak strain transforming; AP, activator protein; APC, adenomatous polyposis coli; cAMP, 3′ 5′‐cyclic adenosine monophosphate; CK1, casein kinase 1δ/1ε; Crb, crumbs; DVL, dishevelled segment polarity protein; EGFR, epidermal growth factor receptor; FAK, focal adhesion kinase; FGFR, fibroblast growth factor receptor; FRMD, FERM and PDZ domain containing; GSK, glycogen synthase kinase; HH, hedgehog; KLF, Krüppel‐like factor; LRP, LDL receptor‐related protein; MAP4K, mitogen‐activated protein kinase kinase kinase kinase; MLC, myosin light; MLCK, myosin light chain kinase; MLCP, myosin light chain phosphatase; NECD, Notch extracellular domain; P, phosphorylation; PDK, pyruvate dehydrogenase kinase; PI3K, phosphoinositide 3‐kinase; PKA, protein kinase A; PP1, protein phosphatase 1; PTPN, protein tyrosine phosphatase non‐receptor type; RASSF, RAS association domain family; ROCK, Rho‐associated kinase; RUNX, Runt‐related transcription factor; SFK, SRC‐family kinase; SMAD, suppressor of mothers against decapentaplegic; SMO, smoothened; STAT, signal transducer and activators of transcription; TAO, thousand and one; TBX, T‐box transcription factor; TGF, transforming growth factor; Ub, ubiquitylation; VEGFR, vascular endothelial growth factor receptor. Dotted lines indicate post‐translational modification events. Figure graphics were created with BioRender.com.

The Hippo pathway is a highly conserved signalling pathway that was first characterized in *Drosophila melanogaster* for its role in larval growth and was later implicated in human cancers as a major tumour suppressor pathway.^42^, ^43^, ^44^, ^45^ The pathway (Figure 2) consists of a core kinase cascade beginning with MST1 (Ste20‐like kinase 1; also known as STK4) and MST2 (also known as STK3), which phosphorylate and activate large tumour suppressor kinases LATS1 and LATS2.^42^, ^44^, ^45^ MST1/2 are activated either by TAO1/2/3 kinase‐mediated phosphorylation, or by trans‐autophosphorylation, of their activation loop.^42^, ^44^ Active MST1/2 phosphorylate Salvador homologue 1 (SAV1) and MOB (monopolar spindle‐one‐binder proteins) kinase activator 1A and 1B (MOB1A/MOB1B), two scaffold proteins that assist MST1/2 in the recruitment and phosphorylation of LATS1/2.^45^, ^46^, ^47^, ^48^, ^49^, ^50^ Two groups of MAP4Ks (mitogen‐activated protein kinase kinase kinase kinase), MAP4K1/2/3/5 and MAP4K4/6/7, work in parallel to MST1/2 and can also directly phosphorylate and activate LATS1/2.^51^, ^52^ Another important player in the Hippo pathway is neurofibromatosis type 2 (NF2)/Merlin, which directly interacts with LATS1/2 and facilitates LATS1/2 phosphorylation by the MST1/2–SAV1 complex.^53^ Upon phosphorylation at their hydrophobic motif by upstream kinases, LATS1/2 subsequently undergo autophosphorylation and are activated.^44^, ^49^, ^50^ In addition to LATS1/LATS2, nuclear Dbf2‐related kinases NDR1 (STK38) and NDR2 (STK38L) also function as YAP/TAZ kinases.^54^ NDR1/2 kinases phosphorylate the paralogous transcriptional co‐regulators YAP and TAZ at consensus HXRXXS motifs, of which YAP has five and TAZ has four.^55^, ^56^, ^57^ The most relevant phosphorylation sites that keep YAP/TAZ inhibited are S127 and S381 in human YAP, and S89 and S311 in human TAZ.^42^, ^43^, ^44^ Phosphorylation at different residues can regulate independent fates of YAP/TAZ. LATS1/LATS2‐mediated YAP‐S127 and TAZ‐S89 phosphorylation creates a binding site for 14‐3‐3 proteins which contribute to keeping YAP/TAZ in the cytoplasm and therefore transcriptionally inactive.^55^, ^56^, ^58^ However, in many cellular contexts this signalling input alone does not appear to be sufficient to keep YAP/TAZ in the cytoplasm, as S127/S89‐phosphorylated YAP/TAZ have also been detected in the nucleus.^59^, ^60^, ^61^, ^62^ Consequently, nuclear localization alone may not always be a reliable surrogate of YAP/TAZ activity, since mice carrying a Yap^S112A^ (similar to S127 in human YAP) knock‐in mutation are surprisingly without phenotype despite nuclear localization of the mutant YAP protein.^63^ Indeed, feedback activation of Hippo signalling was found to ensure physiological levels of YAP/TAZ activity in mammalian cells.^63^, ^64^, ^65^ S381/S311 phosphorylation of YAP/TAZ primes a phosphodegron that can be further phosphorylated by CK1δ/ε to recruit the SCF^β‐TRCP^ E3 ubiquitin ligase complex to tag YAP/TAZ for proteasomal degradation.^55^, ^56^, ^58^ It is worth noting here that YAP/TAZ regulation is not static, but rather dynamic. Indeed, YAP/TAZ undergo constant phosphorylation and dephosphorylation, and are rapidly shuttled between the cytoplasm and the nucleus.^66^, ^67^, ^68^

Importantly, YAP and TAZ are transcriptional co‐regulators that do not contain DNA‐binding domains.^69^ The primary transcriptional binding partners are transcription factors (TF) of the TEA domain family (TEAD1‐4).^43^, ^70^, ^71^ In complex with a TEAD TF, YAP/TAZ bind to gene enhancer elements, and interact with chromatin remodelling factors and modulate RNA polymerase II activity to drive or repress the expression of target genes, which prominently include cell cycle, cell migration and cell fate regulators.^12^, ^72^, ^73^, ^74^, ^75^, ^76^, ^77^, ^78^ YAP/TAZ transcriptional activity is negatively modulated by VGLL4.^79^, ^80^ While TEAD TFs are the predominant transcriptional interaction partners of YAP/TAZ, they have also been shown to physically interact with other TFs such as p63, p73, TBX5, RUNX1/2/3, KLF4 and STAT3.^60^, ^77^, ^81^, ^82^, ^83^, ^84^, ^85^, ^86^, ^87^, ^88^ YAP‐TEAD complexes appear to cooperate closely with other TFs, most notably those of the AP1 family.^12^, ^76^, ^89^, ^90^ YAP and TAZ appear to be have overlapping and non‐redundant roles,^69^, ^91^ since evidence has accumulated that both paralogues might drive distinct transcriptomes.^87^, ^92^, ^93^, ^94^

The Hippo signalling pathway receives myriads of inputs from several intracellular and extracellular cues, which form a complex network to regulate YAP/TAZ localization, abundance and activity (Figure 2).^42^, ^43^, ^44^, ^70^ In most scenarios, these signalling cues modulate the core kinase cascade by relaying signals from the plasma membrane. However, only few dedicated transmembrane receptors and extracellular ligands of the Hippo pathway have been identified (Figure 2). Instead, most upstream signalling components have roles in other processes such as the establishment of cell morphology,^61^, ^95^, ^96^, ^97^, ^98^, ^99^ cell–cell and cell‐matrix adhesion,^13^, ^62^, ^100^, ^101^, ^102^, ^103^, ^104^, ^105^, ^106^, ^107^, ^108^ and cell polarity.^109^, ^110^, ^111^, ^112^, ^113^, ^114^, ^115^, ^116^, ^117^ In addition, there are several proteins that directly regulate YAP/TAZ localization and activation without affecting LATS or NDR kinase activities, such as for example ASPP2/PP1 and PTPN14.^42^, ^43^, ^70^, ^118^ Hippo signalling is highly sensitive to mechanical cues including cell density, mechanical stress, ECM stiffness and ECM composition, which regulate YAP/TAZ through changes in cell geometry and cytoskeleton confirmation and tension.^42^, ^70^, ^119^

The Hippo signalling pathway is also modulated by extensive crosstalk with other signalling pathways.^42^, ^43^, ^44^, ^45^ These include signalling through G protein‐coupled receptors (GPCRs), activated by either lipids (lysophosphatidic acid and sphingosine‐1‐phosphophate) or hormones (glucagon or adrenaline)^120^, ^121^, ^122^, ^123^; the WNT pathway, which can regulate YAP/TAZ either through incorporation into the β‐catenin destruction complex or through destruction complex‐independent mechanisms^124^, ^125^, ^126^, ^127^, ^128^, ^129^; SRC family kinases that promote YAP/TAZ nuclear localization and transcriptional activity either directly by phosphorylating tyrosine residues or indirectly by repressing LATS1/LATS2^13^, ^62^, ^129^, ^130^, ^131^, ^132^, ^133^, ^134^; TGF‐β signalling, which regulates YAP nuclear translocation by targeting the Hippo pathways scaffold RASSF1^135^; the PI3K pathway, which either modulates the core Hippo cascade via PI3K‐PDK1 or YAP/TAZ localization via AKT‐mediated phosphorylation^13^, ^102^, ^136^, ^137^, ^138^; the NOTCH pathway, which modulates YAP/TAZ levels and activity^139^, ^140^, ^141^ and the HH pathway which controls nuclear abundance of YAP/TAZ.^142^, ^143^

## 5 THE ROLES OF HIPPO/YAP/TAZ SIGNALLING IN SKIN HOMEOSTASIS AND REPAIR

There is now substantial evidence for the importance of YAP/TAZ in driving epidermis homeostasis and repair. Consistent with the predominantly nuclear localization of YAP/TAZ in SC‐containing compartments during HF growth,^13^, ^123^, ^144^, ^145^, ^146^ tamoxifen‐induced depletion of *Yap* and *Taz* in Krt5‐expressing epidermal stem/progenitor cells (*K5‐CreERT*/*Yap*/*Taz*) of adult mice led to progressive hair loss beginning 2 weeks after the first tamoxifen injections, while only causing a moderate reduction of basal cell proliferation in the IFE.^13^ This appears to be consistent with the reduced nuclear localization of YAP (and TAZ) in the basal epidermal cell layer of adult compared to foetal and neonatal mice.^13^, ^123^, ^144^ However, conditional knockout of *Yap*/*Taz* in adult epidermis significantly impaired epidermal tissue repair upon skin wounding,^13^ similar to topical treatment of skin wounds with YAP‐interfering RNAs.^147^ Likewise, YAP/TAZ were found to be required for promoting stem/progenitor cell cycling in the IFE in response to mechanical stretching of the epidermis.^148^ Mice lacking the YAP/TAZ co‐factor WBP2 did not have hair growth abnormalities but displayed reduced proliferation in the regenerating epidermis in response to skin wounding.^59^ The roles of YAP/TAZ in murine epidermis remain somewhat ambiguous, since two studies reported no obvious skin phenotypes in epidermis‐specific conditional *Y* *a* *p*/*T* *a* *z* double knockout mice.^12^, ^14^ This discrepancy can likely be explained by the use of different promoters to drive conditional Cre transgene expression (bovine *Krt5* promoter^13^ vs. human *KRT14* promoter^12^, ^14^), which are known to have different deletion efficiencies and onsets/timings. Studies using a Cre‐inducible *LacZ* reporter have indeed found that *K5‐Cre* drives efficient recombination in both IFE and HFs, whereas very little recombination activity could be detected in HFs of *K14‐Cre* mice.^25^, ^149^, ^150^ The conflicting reports of the consequences of conditional *Y* *ap*/*T* *az* knockout in the adult epidermis are thus reminiscent of previous studies on the in vivo functions of ITG beta 1(ITGB1) in the epidermis.^150^, ^151^ Other factors explaining the disparities in *Yap*/*Taz* knockout phenotypes could be tamoxifen dosage and treatment regimens and different genetic backgrounds and age of the mice used (e.g. Elbediwy et al.^13^ used intraperitoneal (ip) injections of 0.1 mg tamoxifen/g body weight in 8‐ to 16‐week‐old mice over 8 weeks, three times per week; the study from Zanconato et al.^12^ used 6‐ to 8‐week‐old mice and three ip injections of 1 mg tamoxifen per week over two weeks; Debaugnies et al.^14^ used P28 mice and 2.5 mg tamoxifen/injection for four consecutive days to knock‐out *Y* *ap* and *T* *az*), and general differences in the animal colonies (food, air and water) leading to different metabolism and microbiota. Of note, in tamoxifen‐induced *K5‐CreERT*/*Yap*/*Taz* epidermis, basal cells that escaped Cre‐mediated recombination were found to be able to repopulate the mutant tissue in a short time frame,^13^ which could have further complicated data interpretation in the different *Y* *ap*/*T* *az* knock‐out studies. That said, human *KRT14* or bovine *Krt5* promoter‐driven expression of mutant, hyperactive YAP transgenes with enhanced nuclear localization (*YAP‐S127A* ^100^ or *NLS‐YAP‐5SA* ^152^ hereafter referred to as K14/YAP‐S127A and K5/NLS‐YAP‐5SA, respectively), in stem/progenitor cells of adult murine epidermis caused severe tissue dysplasia as a consequence of increased stem/progenitor cell proliferation and loss of terminally differentiated cell types, ultimately leading to the formation of cSCC‐like tumors.^100^, ^144^, ^152^ In contrast, mice expressing a different YAP transgene (*YAP‐5SA‐DC*, lacking the C‐terminal transactivation domain) under control of the bovine *Krt5* promoter developed only a mild skin phenotype.^145^, ^146^ In such mice, hyper‐thickening of the IFE resulted from expansion of both the basal and suprabasal cell compartments as well as hyperkeratinization in the most differentiated cell layers.^145^ This suggests that the C‐terminus of YAP may control the balance between epidermal stem/progenitor cell proliferation and differentiation in the IFE. Using cultured human keratinocytes and mice expressing a genetically encoded inhibitor of the interaction of YAP and TAZ with TEADs, recent studies identified a regulatory loop whereby YAP/TAZ/TEADs and KLF4, a TF involved in promoting terminal differentiation,^153^ limit each other's activities to balance proliferation and differentiation.^77^, ^89^ However, these studies did not reveal a direct role of YAP/TAZ/TEAD‐mediated transcription in the regulation of terminal differentiation.^77^, ^89^ RNAi‐mediated silencing of TEAD expression was also found to impair proliferation of primary mouse and human keratinocytes in culture, highlighting that TEADs might be the predominant transcriptional interaction partners of YAP/TAZ in the epidermis.^59^, ^144^

Human keratinocytes require sophisticated culture conditions to maintain their full regenerative potential. A cell culture method developed in the mid‐1970s^154^ is still regarded as “gold standard” in regenerative medicine settings. Under these culture conditions, the capacity of individual keratinocyte colonies to generate secondary cultures can be quantified via morphological and functional clonal analysis.^155^ Based on this procedure, founder colonies (clones) can be categorized as holoclones, meroclones and paraclones.^155^ Holoclones possess the greatest (long‐term) proliferative potential and self‐renewal ability; paraclones are colonies with short lifespan where most cells have committed to undergo terminal differentiation, and meroclones have intermediate properties.^155^ Clonal tracing of human transgenic epidermis revealed that in situ the holoclone‐forming keratinocytes are indeed self‐renewing, long‐lived SCs, which maintain the epidermis long‐term and give rise to pools of short‐lived progenitors (meroclones and paraclones) that ultimately replenish differentiated cells and contribute to wound healing.^19^ Ablation of YAP/TAZ was shown to selectively deplete holoclones and impair regeneration of human epidermal tissue in 3D organotypic skin equivalents,^59^, ^105^ while enforced YAP expression prevented conversion of SCs into progenitors and indefinitely extended the culture lifespan.^105^ YAP expression is dramatically decreased in Junctional Epidermolysis Bullosa (JEB) keratinocytes, which contain only cytoplasmic YAP.^105^ This could explain the slow but progressive loss of JEB patient's ability to heal their continuously occurring skin blisters.^105^

**FIGURE 3:**
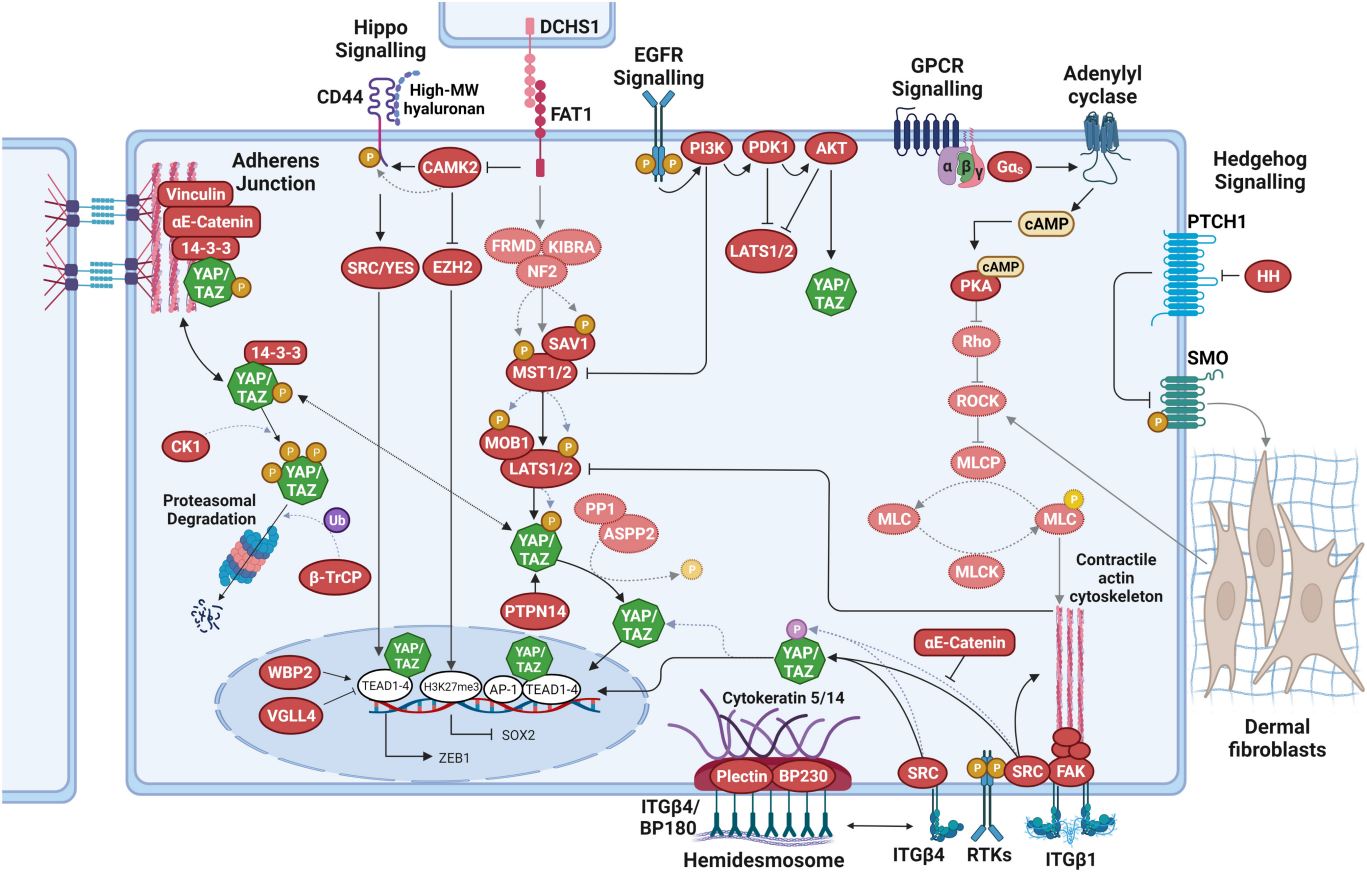
Regulation of YAP/TAZ in normal and neoplastic epidermal cells. Speculative aspects of signalling pathways that are not yet supported by experimental data are indicated by faded graphical elements. Hippo signalling via MOB1A/MOB1B and large tumour suppressor (LATS)1/LATS2 inhibits Yes‐associated protein (YAP) and transcriptional co‐activator with PDZ‐binding motif (TAZ) via serine phosphorylation (orange) to promote cytoplasmic retention and/or proteasomal degradation. Integrin (ITG)–SRC signalling in the basal epidermal cell compartment promotes YAP/TAZ nuclear localization and TEA domain (TEAD) binding. Direct phosphorylation of YAP/TAZ on tyrosine residues (pink) by SRC promotes increased nuclear localization. In the nucleus, vestigial such as family member 4 (VGLL4) competes with YAP/TAZ in binding to TEADs, while WW domain binding protein‐2 (WBP2) enhances the co‐activator functions of YAP/TAZ. The contractile F‐actin‐myosin cytoskeleton stabilizes ITGβ1 adhesions and thus contributes to SRC activation. ITGβ4 adhesions are part of hemidesmosomal complexes that are anchored to keratin 5/14 intermediate filaments. Nuclear localization of YAP in basal epidermal cells is also negatively regulated by PTPN14. At adherens junctions, α‐catenin controls YAP/TAZ activity and phosphorylation by modulating its interaction with 14–3‐3. α‐catenin can also inhibit activation of SRC by ITGβ4. EGFR signalling inactivates the Hippo pathway through the stimulation of PI3K–PDK1. G protein‐coupled receptor (GPCR) signalling, involving Gαs and PKA, suppresses LATS1/2 activation, presumably via decreasing F‐actin‐myosin cytoskeletal contractility downstream of Rho/ROCK. During cSCC progression, loss of function of the protocadherin FAT1 activates a CAMK2‐CD44‐SRC axis that promotes nuclear translocation of YAP and this drives the expression of zinc finger E‐box binding homeobox 1 (ZEB1) that stimulates the mesenchymal state. FAT1 loss of function also inactivates enhancer of zeste homologue 2 (EZH2), promoting SRY‐box transcription factor 2 (SOX2) expression, which sustains the epithelial state. Together, these molecular events promote a hybrid epithelial‐to‐mesenchymal transition (EMT) phenotype. If FAT1 can directly activate the Hippo pathway, is currently not known. In BCC, fibroblast activation and ECM remodelling in papillary dermis as a consequence of increased HH signalling in the epidermis may indirectly activate epidermal ROCK signalling through mechano‐reciprocity. AKT, Ak strain transforming; AP, activator protein; cAMP, 3′ 5′‐cyclic adenosine monophosphate; CAMK, Ca^2+^/calmodulin‐dependent protein kinase; CK1, casein kinase 1δ/1ε; DCHS, dachsous; EGFR, epidermal growth factor receptor; FAK, focal adhesion kinase; HH, hedgehog; MLC, myosin light chain; MLCK, myosin light chain kinase; MLCP, myosin light chain phosphatase; P, phosphorylation; PDK, pyruvate dehydrogenase kinase; PI3K, phosphoinositide 3‐kinase; PKA, protein kinase A; PP1, protein phosphatase 1; PTPN, protein tyrosine phosphatase non‐receptor type; RASSF, RAS association domain family; ROCK, Rho‐associated kinase; SFK, SRC‐family kinase; SMO, smoothened; Ub, ubiquitylation. Dotted lines indicate post‐translational modification events. Figure graphics were created with BioRender.com.

While YAP/TAZ clearly play important roles in promoting stem‐ and progenitor cell self‐renewal during HF cycling and epidermal tissue repair, there is still ambiguity as to what extent the Hippo signalling pathway is involved in controlling the activity of YAP/TAZ in the epidermis.^8^ While epidermis‐restricted (*K14‐Cre*) conditional knockout of *Mst1*/*Mst2* during mouse development was without consequence for tissue homeostasis and Yap activity even in adult animals,^100^ conditional tamoxifen‐induced (*K14‐CreER*) double knockout of *Mob1a* and *Mob1b* in postnatal mouse epidermis led to a marked expansion of the stem/progenitor cell populations through increased nuclear Yap abundance^156^ (Figure 3), reminiscent of the epidermal phenotypes of K14/YAP‐S127A and K5/YAP‐5SA‐DC transgenic mice.^100^, ^144^, ^145^ The consequences of epidermis‐restricted LATS1/LATS2 knockout have not been studied yet. However, phosphorylation of LATS1/LATS2 in response to activation of upstream kinases^156^ and increased Yap transcriptional activity upon RNAi‐mediated *Lats1*/*Lats2* ablation^130^ in mouse keratinocytes support a role of LATS1/LATS2 in controlling YAP/TAZ in mouse epidermis (Figure 3). Moreover, deletion of *Gnas* (the gene coding for the Gαs heterotrimeric G‐protein) or inactivation of protein kinase A (*Pka*) in mouse epidermis (*K14‐CreER*) led to aberrant expansion of the stem/progenitor cell compartment through activation of YAP, likely involving LATS1/2 but not MST1/2^123^ (Figure 3). Nuclear localization of YAP in basal epidermal cells appears to also be negatively regulated by PTPN14^157^ (Figure 3).

It is interesting to note that *L* *ats*1/*L* *ats*2 knockdown in human keratinocytes affected YAP nuclear localization and transcriptional activity only in confluent, fully contact‐inhibited cultures, indicating that efficient control of YAP/TAZ localization and activity likely involves integration of multiple signalling cues, in particular mechanical cues.^59^, ^95^ Indeed, the adherens junction component αE‐catenin has been identified as a cell density‐dependent YAP regulator in several studies.^100^, ^101^, ^106^, ^130^ Genetic deletion of αE‐catenin in murine epidermis (*K14‐Cre*) or more specifically in the HF bulge (*GFAP‐Cre*) led to epidermal hyperproliferation, associated with increased nuclear abundance of YAP.^100^, ^101^ Several mechanisms have been proposed of how αE‐catenin regulates YAP/TAZ. In one mechanism, αE‐catenin promotes YAP S127 phosphorylation and cytoplasmic localization directly by regulating its interaction with 14‐3‐3 proteins^100^(Figure 3). A more recent study found that in adherens junctions, vinculin keeps αE‐catenin in mechanically engaged, stretched/open conformation. The stretched αE‐catenin molecules create binding sites for 14‐3‐3 proteins, which sequester pS127‐YAP at the adherens junctions.^106^ This mechanism appears to be conserved also in human keratinocytes, where disruption of actin‐myosin‐mediated cytoskeletal tension at adherens junctions leads to nuclear re‐entry of YAP in contact‐inhibited cultures.^59^

In a second mechanism, αE‐catenin suppresses SRC family kinase (SFK)‐mediated tyrosine phosphorylation of YAP, thereby preventing YAP's nuclear localization and TEAD binding^130^ (Figure 3). Interestingly, both αE‐catenin‐dependent mechanisms appear to operate independently of LATS1/LATS2.^100^, ^130^ Integrins not only provide epidermal stem/ progenitor cell markers, but they also regulate stem and progenitor cell fate during homeostasis, tissue repair and cancer progression.^158^, ^159^ Accordingly, in cultured human keratinocytes, inhibition of ITGB1 or of its downstream effectors SRC and FAK or PI3K impaired YAP/TAZ nuclear localization.^13^, ^138^ Likewise, epidermis‐restricted deletion of SRC or FAK, or pharmacological inhibition of SFK activity, led to decreased YAP levels and nuclear localization in basal keratinocytes^13^ (Figure 3). ITGB1‐mediated activation of YAP appears to depend on the integrity and organization of the F‐actin cytoskeleton,^13^, ^70^ but less so on actomyosin contractility.^13^, ^59^ In addition, the hemidesmosome‐associated ITGB4^160^ was also shown to control YAP activity via direct SRC‐mediated phosphorylation of YAP,^130^ which is negatively regulated by αE‐catenin.^130^

## 6 YAP AND TAZ AS ONCOPROTEINS IN SKIN CANCERS

YAP/TAZ are overexpressed in many different types of murine and human cancers.^9^, ^161^, ^162^, ^163^, ^164^ Of note, YAP/TAZ appear to be particularly important in squamous cell cancers, which are characterized by frequent amplification of YAP/TAZ.^161^ YAP and TAZ are highly expressed and nuclear in different types of BCC in both human and mice.^14^, ^59^, ^146^, ^165^, ^166^ In cSCC, increased YAP expression was shown to correlate with disease progression.^14^, ^152^, ^167^ In contrast, TAZ expression appears to be more sparse in cSCC, with fewer cells staining positive for nuclear TAZ.^14^ Increased nuclear YAP expression was also observed in a subset of kerathoacanthomas with low αE‐catenin expression.^101^ YAP overexpression was also documented in pilomatrixoma and trichilemmal carcinoma, rare tumours of HFs.^156^, ^168^

In accordance with their increased expression in keratinocyte cancers, YAP and TAZ were found to play key roles in the development and progression of cSCC. Several studies found YAP/TAZ to be essential for in vitro proliferation of human cSCC cell lines, by promoting G1/S progression.^35^, ^59^, ^167^ Expression of oncogenic *Kras* ^*G12D*^ (together with *Tp53* knockout) in HF SCs using *Lgr5‐CreER*/*KrasG12D*/*Tp53*KO mice induces cSCCs with varying degrees of squamous differentiation.^14^ In this cSCC model, *Yap*/*Taz* deletion completely abrogated tumour formation due to rapid cell death of the oncogene‐expressing cells.^14^ In a chemical two‐stage skin carcinogenesis mouse model, which involves tumor initiation by the application of a sub‐carcinogenic dose of a carcinogen (e.g. 7,12‐dimethylbenz[a]‐anthracene (DMBA)) and subsequent tumour development by repeated treatment with the tumour‐promoting agent 12‐O‐tetradecanoylphorbol‐13‐acetate (TPA),^169^ YAP/TAZ were also shown to be essential for tumor development.^12^ Conversely, development of cSCC‐like tumors was observed in K14/YAP‐S127A and K5/NLS‐YAP‐5SA mice,^100^, ^152^ the latter displaying progression to spindle cell carcinoma at sites of scratch wounding where YAP‐mediated activation of the TF ZEB1 induced an EMT programme.^152^ Extending these findings, a recent study found that deletion of the protocadherin *Fat1* in a mouse model of cSCC promoted a hybrid EMT phenotype by inducing YAP nuclear translocation and ZEB1 expression that stimulates the mesenchymal state, while increased expression of the cancer SC factor SOX2^170^ sustains the epithelial state^35^ (Figure 3). A role of YAP in cSCC progression is also supported by in vitro studies demonstrating YAP functions in cell migration and invasion.^167^, ^171^ Conversely, overexpression of the YAP/TAZ negative regulator VGLL4 was found to reduce growth of human cSCC cells^172^ (Figure 3). In human keratoacanthomas and cSCC, strong correlation between low αE‐catenin abundance and nuclear YAP localization has been documented.^100^, ^101^, ^130^ Accordingly, conditional knockout of αE‐catenin in the HF bulge was shown to cause development of keratoacanthomas displaying increased nuclear YAP abundance.^101^

Similar to the situation in genetically induced cSCC, epidermis‐restricted deletion of *Yap*/*Taz* in a mouse model of BCC (driven by mutant SMO; *K14CreER*/*SmoM2* mice), efficiently prevented tumour initiation.^14^ A different study found that conditional deletion of *Yap*, but not *Taz*, significantly reduced the tumour burden of *K14CreER*/*SmoM2* mice while not completely abrogating BCC formation.^165^ This suggests that in murine BCC YAP is the dominant paralogue.^165^ Importantly, clonal tracing of induced BCC tumours demonstrated that Yap‐null clones had a decreased fitness, initially becoming outcompeted by YAP‐positive clones and ultimately becoming depleted as the tumours progressed to an invasive phenotype.^165^ Consistent with these findings, inhibition of YAP/TAZ‐Tead binding was found to lead to rapid elimination of tumour cells in BCC lesions.^89^ There is also evidence that in BCC, activation of oncogenic HH signalling in the epidermis may be closely linked to activation of Rock‐dependent mechano‐signalling in the dermal stroma, potentially leading to positive feedback activation of YAP/TAZ^143^ (Figure 3).

YAP promotes BCC initiation and progression via TEAD TFs to drive JNK‐Jun signalling both at the level of c‐Jun gene transcription but also upstream of c‐Jun by controlling JNK activation.^165^ c‐Jun is a component of the functionally diverse AP‐1 TF complex, and in several cell types, YAP/TAZ/TEAD and AP‐1 were shown to cooperate to drive the expression of target genes involved in the cell cycle control of S‐phase entry and mitosis.^8^, ^12^, ^76^, ^77^ Indeed, co‐occupation of chromatin regions by TEADs and AP‐1 TFs was observed also in normal keratinocytes and BCC by ChIP sequencing analysis.^77^, ^89^, ^165^ However, recent findings suggest that YAP/TAZ regulate inflammation‐related gene networks in BCC also independently of TEAD.^89^ If cSCC initiation and progression also depends on cooperation of TEADs with AP1 factors remains to be firmly established. Of note, impaired tumour formation in the two‐stage skin carcinogenesis model, where TPA treatment activates AP1 TF complexes, does indeed suggest AP1‐YAP/TAZ/TEAD cooperation in driving cSCC development.^12^

Several studies found YAP/TAZ expression to be elevated in most benign and dysplastic nevi and in situ CM, however, without significant differences between lesion types.^173^, ^174^, ^175^ Moreover, none of these studies observed striking fluctuations in the YAP/TAZ nuclear/cytoplasmic ratio (which is often used as a proxy for YAP/TAZ activity) in different stages of CM development.^173^, ^174^ However, using YAP/TAZ target gene expression as a more robust read out of YAP/TAZ activity, a recent study discovered that YAP/TAZ activity was elevated in invasive CM.^176^ This is consistent with an unbiased transcriptomics study of human CM tissues, which revealed TEAD TFs as regulators of the invasive cell state.^177^ Interestingly, although BRAF inhibitor‐resistant melanoma cells were shown to depend on YAP/TAZ for their proliferation and survival,^178^ YAP/TAZ activity is not associated with the mutation status of BRAF and NRAS.^176^ This is consistent with findings that YAP/TAZ sensitivity in CM cells does not correlate with either BRAF or NRAS mutation status.^174^ Of note, somatic hypermutations of *YAP* *1* were also detected in CM, but so far only in a single patient.^174^ These YAP mutations manifested as seven serine to alanine transpositions. Since four of these serines are the key regulatory residues phosphorylated by the core Hippo pathway kinases LATS1/LATS2 and NDR1/NDR2,^42^, ^43^, ^44^ the mutant YAP allele was consequently shown to code for a hyperactive YAP protein.^174^ While the increased expression of YAP/TAZ in non‐invasive CM could suggest a role in tumour development, studies using patient‐derived CM xenografts and established CM cell lines revealed only a variable requirement of YAP/TAZ/TEAD for cell viability.^174^ In contrast, important roles for YAP/TAZ in invasive CM and metastasis have been clearly demonstrated.^173^, ^176^ The mechanism by which YAP/TAZ activity is promoted in invasive melanoma cells compared with non‐invasive cells is currently unclear.^174^, ^176^

## 7 TARGETING YAP AND TAZ FOR SKIN CANCER TREATMENT

In the clinic, the biggest challenge for skin cancers remains treatment of patients with advanced or metastatic disease.^3^, ^4^, ^8^, ^29^, ^36^, ^179^, ^180^ There are several excellent comprehensive reviews on current treatment options.^5^, ^180^, ^181^, ^182^ Since Hippo signalling acts as a tumor suppressor pathway and aberrant YAP/TAZ activity is implicated in various types of skin cancers, targeting Hippo/YAP/TAZ signalling offers potential opportunities for cancer therapy. Below, we highlight current strategies for therapeutic intervention, some of which are showing promise in initial pre‐clinical studies.

### 7.1 Targeting YAP/TAZ nuclear shuttling

As transcription co‐regulators, YAP/TAZ exert their activity in the nucleus. Consequently, interfering with the nuclear localization YAP/TAZ could be one approach to inhibit YAP/TAZ activity (Table 1). The photosensitizer verteporfin was found to have significant anti‐tumour effects.^183^, ^184^ Several reports have indicated that verteporfin can block YAP‐TEAD activity, either through disruption of YAP‐TEAD binding,^185^ or through increased cytoplasmic sequestration of YAP by 14–3‐3σ.^186^ However, verteporfin‐mediated anti‐tumor effects may not be specific to only inhibiting YAP‐TEAD complexes, as verteporfin has also been reported to have proteotoxic effects.^187^, ^188^, ^189^ A35, a synthetic inhibitor of DNA topoisomerase II, was found to decrease YAP nuclear localization by activating its phosphorylation.^190^ Likewise, dichloroacetate, a small molecule metabolic regulator used for treating mitochondrial genetic diseases and lactic acid poisoning, was shown to promote the nuclear‐cytoplasmic translocation of YAP.^191^ However, as in the case of verteporfin, the reported anti‐tumour effects of these compounds are likely not YAP/TAZ‐specific.

**TABLE 1:** YAP/TAZ nuclear shuttling inhibitors

Molecule	Other name(s)	Inhibitory action	Binding validation	Activity validation	Binding affinity (K_D_)	IC50	Reference(s)	Vendor
Verteporfin	Visudyne	Inhibitor of YAP nuclear localization	NR	Co‐IP, TEAD luciferase reporter, PLA, YAP‐TEAD target gene qPCR	NR	8 nM–11.16 μM (Cell line‐dependent)	184, 185, 195	Selleckchem (#S1786) Tocris (#5305)
A35	CHEMBL3041188	YAP S127 phosphorylation agonist	NR	WB	NR	<1 μM (Cell line‐dependent)	^190^	
Dichloroacetate	Dichloroacetic acid salt	Inhibitor of YAP nuclear localization (Hippo‐dependent)	NR	IFM, WB, RNA‐seq, TEAD luciferase reporter, YAP‐TEAD target gene qPCR	NR	80–183 μM	^191^	Selleckchem (#S8615)
Dasatinib	Sprycel	Inhibitor of YAP nuclear localization (tyrosine kinase dependent)	NR	IFM, TEAD luciferase reporter, WB, YAP‐TEAD target gene qPCR	NR	~1 nM	^130^	Selleckchem (#S1021) Merck (#SML2589)
Pazopanib		YAP phosphorylation agonist and promotes proteasomal degradation	NR	IFM, TEAD luciferase reporter, WB	NR	<15 μg/ml	^199^	Merck (#SML3104)
CA3	CIL56	Inhibitor of YAP expression	NR	IFM, TEAD luciferase reporter, WB, YAP‐TEAD target gene qPCR	NR	<1 μM (Cell line dependent)	194, 195	Selleckchem (#S8661)
Statins: Cerivastatin Mevastatin Simvastatin Fluvastatin	Brand names: Baycol Compactin Zocor Lescol	Inhibitors of YAP nuclear localization via mevalonate pathway (HMG‐CoA reductase)	NR	IFM, TEAD luciferase reporter, WB, YAP‐TEAD target gene qPCR	NR	<10 μM ~26 nM ~6 μM >0.8 μM (Cell line‐dependent)	198, 199, 200, 239	Merck (#SML0005) (#M2537) (#S6196) (#SML0038)
PI3K‐AKT/PDK1 signalling inhibitors: LY294002 – PI3Ki AKT inhibitor V – AKTi PDK1 inhibitor II – PDK1i Wortmannin – PI3Ki AKT inhibitor VIII – AKTi Calphostin C – PKCi Gö 6983 – PKCi BX795 – PDK1i, TBK1i	Triciribine	YAP phosphorylation agonists via PDK1‐Hippo‐dependent activity	NR	IFM, YAP‐TEAD target gene qPCR	NR	<10 μM <20 μM 6 nM 2–4 nM 0.058–2.12 μM 50 nM 6–60 nM 6 nM	136, 138, 204	Merck (#L9908) (#124038) (#521276) (#W1628) (#124017) (#C6303) (#G1918) Santa‐Cruz (#sc‐281 689)

Abbreviations: Co‐IP, co‐immunoprecipitation; IFM, immunofluorescence microscopy; NR, not reported; PLA, proximity ligation assay; WB, Western blotting.

Dasatinib, a pharmacological inhibitor of SFKs, could have potential as a YAP‐targeting therapy for cSCC. In orthotopic mouse xenograft models, dasatinib treatment caused prominent inhibition of tumour growth through interference with SFK‐induced YAP activation.^130^ Of note, topical application of dasatinib onto murine cSCC was found to induce tumour regression with less side effects when compared to treatment with 5‐fluorouracil, one of the standard chemotherapies for cSCC.^192^ Suppression of YAP/TAZ activity by SFK inhibition might also be beneficial for the treatment of BRAF inhibitor‐resistant metastatic melanomas, in which YAP‐induced PD‐L1 expression drives immune evasion.^131^, ^193^

CA3, a small molecule with anti‐tumour activity in various cancers, notably including head and neck SCC and cSCC, appears to act by reducing YAP expression, probably at the level of *YAP1* gene transcription.^172^, ^194^, ^195^, ^196^, ^197^

Statins, a class of drugs used to lower the level of low‐density lipoprotein (LDL) cholesterol in the blood, block YAP/TAZ nuclear localization and activity through Rho‐GTPases.^198^, ^199^, ^200^ Combined EGFR and YAP inhibition (with statins) prolongs survival in lung cancer patients.^201^ Consequently, a similar targeted approach could potentially be applied to treat invasive BRAF inhibitor‐resistant melanoma with EGFR overexpression^199^, ^202^, ^203^ and YAP/TAZ dependency.^178^, ^193^ Other interesting compounds with documented YAP/TAZ inhibition capacity that could be used for combination therapies (together with SFK or statin inhibitors) are multi‐tyrosine kinase inhibitors such as pazopanip and PI3K‐AKT inhibitors.^136^, ^138^, ^199^, ^204^

### 7.2 Targeting the YAP/TAZ‐TEAD interface

YAP/TAZ are natively unfolded proteins, making it a challenge to target them directly. However, YAP/TAZ become structured upon binding to TEADs.^205^, ^206^, ^207^ This enables targeting of the YAP/TAZ‐TEAD binding interface by protein–protein binding disruptors (PPBDs).^208^ Crystal structures of the YAP‐TEAD complex have revealed that the C‐terminal YAP‐binding domain of the TEADs comprises an immunoglobulin‐like β‐sandwich fold structure and two helix‐turn‐helix motifs.^205^, ^207^, ^209^, ^210^, ^211^ The N‐terminal TEAD‐binding motif of YAP adopts a helix–loop–helix structure, with helix α1 and helix α2 forming the main hydrophobic and hydrogen‐bond interactions with TEADs.^205^, ^207^, ^209^, ^210^, ^211^, ^212^ The loop region in YAP is relatively long and also forms interactions with TEADs. Consequently, TAZ‐TEAD binding differs slightly from YAP‐TEAD binding because of a shorter loop region.^212^ Interestingly, the TAZ‐TEAD crystal structure revealed two possible binding modes of the co‐regulator with its transcription factor (1:1 and 2:2 complexes), with implications for target gene expression.^212^ However, despite these structural differences, PPBDs able to disrupt the YAP‐TEAD interaction should have the ability to also disrupt the TAZ‐TEAD interaction.

The interaction between the N‐terminal motif of YAP/TAZ and the C‐terminal YAP/TAZ‐binding domain in TEADs involves three highly conserved interfaces, with the third interface being the major energetic determinant of high‐affinity binding.^205^, ^207^, ^209^, ^210^, ^211^, ^212^ The crystal structures of YAP‐TEAD complexes revealed clear surface pockets at the second and third interfaces that might enable the rational design of PPBDs^163^, ^208^ (Table 2). Of note, residues within the YAP/TAZ‐binding TEAD pocket at the third binding interface are highly conserved across all TEAD family members,^163^, ^207^, ^210^, ^211^ suggesting that targeting this interface could offer possible pan‐TEAD ligands. Indeed, various efforts to target the second and third YAP‐TEAD interface have yielded promising peptides and small molecules,^208^, ^213^ which are summarized in Table 2. Another pocket in the centre of the C‐terminal YAP‐binding domain of TEADs is also accessible to small molecules.^209^ Palmitate is the natural ligand that binds to the central pocket.^214^, ^215^ Several central pocket binders have been identified that inhibit TEAD palmitoylation,^208^, ^213^ with some of them acting as allosteric PPBDs by disrupting YAP‐TEAD interaction, while others are not (Table 3). The permeability, specificity, efficacy, in vivo impact and, ultimately, safety of all these inhibitors remains to be determined.

**TABLE 2:** YAP/TAZ‐TEAD inhibitors of protein–protein interaction, surface binding

Molecule	Other name(s)	Inhibitory action	Binding validation	Activity validation	Interface	Binding affinity (K_D_)	IC50	References	Vendor
TB1G1/TB1G2	Cysteine‐dense peptide (CDP)	iPPI	Rosetta protein design, mammalian surface display, SPR, Co‐IP, PLA	TEAD luciferase reporter, competitive mammalian surface display	2	TB1G1: 31 ± 2 nM TB1G2: 368 ± 4 pM	NR	^240^	
Fragment 1		iPPI scaffold	Co‐crystal structure	ITC, TEAD luciferase reporter	2	~300 μM	NR	^241^	Chemspace (#CSSB00000239375)
Peptide 17	YAP‐TEAD inhibitor 1	iPPI	Molecular docking, co‐crystal structure, pull‐down assay	Pull‐down assay	3	15 nM	25 nM	^242^	Selleckchem (#S8164) AdooQ (#A15858) APExBIO (#A1149) MedChemExpress (#HY‐P2244)
Peptide 10		iPPI	Co‐crystal structure, SPR	Pull‐down assay	3	289.5 nM	297 nM	^243^	
Peptides 9, 10		iPPI	Co‐crystal structure	TR‐FRET	3	Peptide 9: 25 nM Peptide 10: NR	Peptide 9: 16 ± 5 nM Peptide 10: 9 ± 2 nM	^244^	
TEAD‐binding fragment	MFCD00187673	iPPI scaffold	NMR	ITC	3	77 nM	NR	^245^	Chemspace (#CSC000003413)
Dioxo‐benzoisothiazole Example 22		iPPI	NMR	TEAD luciferase reporter, AlphaLISA®	3	NR	83 nM	^246^	patented: WO2017064277A1
1,2,3‐Triazole‐4‐carbohydrazide derivatives (hit 2)		iPPI	Molecular docking, TSA	MST, TSA, TEAD luciferase reporter, YAP‐TEAD target gene qPCR	3	650 μM	6.5 μM	^247^	MCule (#MCULE‐3696035303) MolPort (# MolPort‐002‐604‐580)
Compound 3.1	Pyrazidol, Pirlindol, Pirlindolum	iPPI	Molecular docking, STD NMR	Co‐IP, TEAD luciferase reporter, pull‐down assay, YAP‐TEAD target gene qPCR	3	~12 μM	33–44 μM (TEAD isoform‐dependent)	^248^	Chemspace (#CSC020600808)
Super‐TDU		iPPI	Molecular modelling	Co‐IP, mutational studies	2&3	NR	57.9 ng/ml	^249^	Selleckchem (#S8554) MedChemExpress (#HY‐P1727)

Abbreviations: AlphaLISA, amplified luminescent proximity homogenous assay (linked immunosorbent assay); Co‐IP, co‐immunoprecipitation; iPPI, inhibitor of protein–protein interaction; ITC, isothermal titration calorimetry; MST, microscale thermophoresis; NMR, nuclear magnetic resonance; NR, not reported; PLA, proximity ligation assay; SPR, surface plasmon resonance; STD NMR, saturation transfer difference nuclear magnetic resonance; TR‐FRET, time‐resolved fluorescence resonance energy transfer; TSA, thermal shift assay.

**TABLE 3:** YAP/TAZ‐TEAD inhibitors of protein–protein interaction, central pocket binding

Molecule	Other name(s)	Inhibitory action	Binding validation	Activity validation	Interface	Binding affinity (K_D_)	IC50	Reference(s)	Vendor
Flufenamic acid	Paraflu, Parlef, Ristogen, Sastridex, Tecramine	API	Co‐crystal structure, molecular modelling, STD NMR	ITC, TEAD luciferase reporter	Central pocket & interface 3 (weak affinity)	73 μM	NR	^209^	Tocris (#4522) Merck (#151300)
Niflumic acid	Donalgin, Niflam, Forenol	API	Co‐crystal structure, molecular modelling, STD NMR	ITC, TEAD luciferase reporter	Central pocket & interface 3 (weak affinity)	28 μM	NR	^209^	Tocris (#4112) Merck (#N0630)
TED‐347 (compound 2)		API—covalent	Co‐crystal structure, molecular docking, molecular dynamics simulation, protein mass spectrometry, FP	FP, Biolayer interferometry, Co‐IP, TEAD luciferase reporter, YAP‐TEAD target gene qPCR	Central pocket	NR	5.9 μM	^250^	Selleckchem (#S8951) MedChemExpress (#HY‐125269)
MYF‐01‐037		API –covalent	Molecular docking	YAP‐TEAD split Gaussia luciferase assay, YAP‐TEAD target gene qPCR	Central pocket	NR	0.8 μM	^251^	Selleckchem (#S8950) Cambridge Bioscience (#B3298‐5)
Non‐fused tricyclic compound 42		NR	NR	TEAD luciferase reporter	Central pocket	NR	<0.1 μM	^252^	Patented: WO2018204532A1
MGH‐CP1		API	Co‐crystal structure	Co‐IP, TEAD luciferase reporter	Central pocket	NR	672–710 nM (TEAD isoform‐dependent)	^253^	Selleckchem (#S9735)
Indole incorporated triazine derivatives (e.g compound 9)		API	Molecular docking, NanoDSF, FP	NanoDSF, FP, YAP‐TEAD target gene qPCR	Central pocket	NR	6.75 μM	^254^	
Dihydropyrazolo pyramidines (e.g. compound 7)		NR	NR	TR‐FRET	Central pocket	NR	18–87 nM (TEAD isoform‐dependent)	^255^	Patented: WO2019232216A1
K‐975	K975	API	Co‐crystal structure, SPR	SPR, pull down assay, YAP‐TEAD target gene qPCR	Central pocket	NR	NR	^256^	MedChemExpress (#HY‐138565)
Kojic acid‐derived Betti bases (e.g. compound 19)		API	FP, whole‐protein ESI–MS spectrometry, NMR	Thiol conjugation assay, FP, cellular thermal shift assay	Central pocket	28 nM	0.2 ± 0.04 μM	^257^	
Compound 2		API	Co‐crystal structure, FP, SPR	FP, TR‐FRET, Co‐IP, SPR	Central pocket	229 nM	31.8 nM	^258^	
DC‐TEADin02	DCTEADin02	API—covalent	Molecular docking, NMR, SPR, mass spectrometry	Pull‐down assay, TEAD luciferase reporter, YAP‐TEAD target gene qPCR	Central pocket	NR	197 ± 19 nM	^259^	MedKoo (#463183)
Quinolinol Q2		API	Molecular docking, molecular dynamics simulation	TEAD luciferase reporter, RNA‐seq	Central pocket	2.6 ± 0.3 μM	2.6 μM	^260^	Hit2Lead (#5926377) Mcule (#MCULE‐5191032439) MolPort (#MolPort‐003‐183‐526)

Abbreviations: API, autopalmitoylation inhibitor; Co‐IP, co‐immunoprecipitation; ESI‐MS, electrospray ionization mass spectrometry; FP, fluorescence polarization; ITC, isothermal titration calorimetry; MST, microscale thermophoresis; NMR, nuclear magnetic resonance; Nano‐DSF, nano differential scanning fluorimetry; NR, not reported; PLA, proximity ligation assay; SPR, surface plasmon resonance; STD NMR, saturation transfer difference nuclear magnetic resonance; TR‐FRET, time‐resolved fluorescence resonance energy transfer; TSA, thermal shift assay.

### 7.3 Targeting the YAP/TAZ‐associated transcriptional machinery

Once in the nucleus, YAP/TAZ execute their biological functions by regulating gene transcription through their association with various TFs, as well as chromatin remodelling protein complexes including the switch/sucrose nonfermentable (SWI/SNF) and nucleosome remodelling and deacetylase (NuRD) complexes (reviewed in^216^). Constitutive nuclear YAP/TAZ expression in cancer cells promotes hyper‐transcription at YAP/TAZ target genes and a dependency on YAP/TAZ‐driven transcriptional programmes.^78^, ^217^ Such transcriptional dependencies are amenable to inhibition with small molecules.^218^

For example, in triple‐negative breast cancer cells, YAP/TAZ‐driven transcriptional addiction is mediated through association with the bromodomain and extraterminal (BET)‐coactivator protein BRD4, and consequently YAP/TAZ pro‐tumorigenic activity was shown to be vulnerable to treatment with the BET inhibitor JQ1.^78^

Gene repressive activity by YAP/TAZ in breast epithelial cells was shown to depend on recruitment of the NuRD complex, which has both nucleosome‐remodelling and histone deacetylase (HDAC) activity, to render chromatin inaccessible.^219^ Therefore, HDAC inhibitors may represent a potential therapeutic avenue for specifically targeting the NuRD‐mediated co‐repressor functions of YAP/TAZ. Of note, the HDAC inhibitor vorinostat is FDA approved for the treatment of cutaneous T‐cell lymphoma and is being investigated for the topical treatment of cutaneous malignancies^220^ and in BRAF^V600E^ melanoma.^221^

The functions of SWI/SNF complexes in squamous cell cancers are ambiguous, as both pro‐tumorigenic and tumour‐suppressive roles have been identified in studies focusing on head and neck SCC.^222^, ^223^, ^224^ A key factor in this puzzle appears to be the SWI/SNF subunit ACTL6A, which, when overexpressed, stoichiometrically assembles into BAF‐type SWI/SNF complexes which then drive chromatin loading of TEAD‐YAP complexes.^222^ Should a similar mechanism operate in cSCC, targeting the catalytic subunits of SWI/SNF complexes could be used as therapeutic strategy.^225^

Cooperation of YAP/TAZ‐TEAD with AP1 TFs has been documented in both BCC and cSCC cells.^12^, ^89^, ^165^ Multiple AP‐1 inhibitors including small molecules and peptides have been assessed in preclinical and clinical trials,^226^, ^227^, ^228^ therefore providing a toolbox to potentially interfere with the YAP/TAZ‐TEAD‐AP‐1 complex.

High resolution mapping of DNA double–strand breaks (DSBs) in cancer and non‐tumorigenic cells revealed a transcription‐coupled DNA damage repair mechanism at oncogenic super‐enhancers.^229^ This mechanism involves high transcriptional activity mediated by YAP‐TEAD and AP‐1 factors, which leads to DNA topoisomerase (TOP1)‐mediated induction of DSBs, which are repaired through the homologous recombination DNA damage repair pathway. Depletion of TEAD4 or RAD51 increased DSBs at RAD51/TEAD4 common binding sites within super‐enhancers and decreased expression of related genes, which are mostly oncogenes.^229^ These findings therefore suggest that, at least in certain cancer types, targeting YAP/TAZ‐TEAD could help to selectively reduce transcription of oncogenes.

## 8 CONCLUSIONS/MAJOR OPEN QUESTIONS

Genetic studies in mice have unequivocally demonstrated that YAP/TAZ are essential for BCC and cSCC initiation and progression.^12^, ^14^, ^152^, ^165^ However, due to the inherent differences between mouse and human skin and limitations of genetic mouse models of skin cancers,^230^, ^231^, ^232^ it remains to be vigorously tested if this is true also in the human skin cancer context. That said, we must also acknowledge that there are limitations to the use of human cells/tissues as the correct physiological context (including an appropriate microenvironment) is difficult (if not impossible) to reproduce in vitro. So far, only few studies have assessed the roles of YAP/TAZ in vitro in human skin cancer models, the majority focusing on melanoma.^35^, ^59^, ^131^, ^167^, ^174^, ^176^, ^178^ Additional human cell/tissue‐focused studies are therefore required to address several open questions and to complement studies done in mice and other animal model systems: (1) Is there genetic evidence for YAP/TAZ hyperactivation in human skin cancers? SCCs of the lung, oesophagus and head and neck display the highest levels of *YAP1* or *WWTR1* gene amplifications across multiple cancer types, often in a mutually exclusive manner.^94^, ^161^ Indeed, a comprehensive survey of the genomic landscape of human cSCC was able to detect evidence of *YAP1* amplification in a small set of in situ and invasive cSCC samples.^32^, ^233^ Another study focusing on BCC detected inactivating mutations in *LATS1* and *PTPN14* in 16% and 23% of BCC samples, respectively, as well as missense mutations in *LATS2* (12% of analysed BCCs).^234^ Clearly, a more targeted analysis of genomic alterations of Hippo/YAP/TAZ components across various skin cancer types is therefore warranted. (2) What are the distinct roles and regulatory mechanisms of YAP/TAZ in skin cancer? As discussed in section 5, the mechanisms controlling YAP/TAZ nuclear localization and transcriptional activity in skin cancer are still poorly understood. The majority of past studies on YAP/TAZ focused on either one of the paralogues or had simply assumed similar functions between them. However, emerging evidence demonstrates that YAP and TAZ have distinct roles where they partner with different transcription factors, drive different transcriptional programs and also modulate the tumour microenvironment distinctively.^69^, ^87^, ^91^, ^94^, ^235^, ^236^ This question can be addressed with comprehensive RNAi or CRISPR‐based gene knock‐down/knock‐out studies, ideally performed across a panel of human skin cancer models and employing 3D organotypic skin cancer models to test for physiological relevance.^237^, ^238^ Such studies could also provide dermatologists with prognostic cSCC and BCC‐specific YAP/TAZ signatures, as exists already for melanoma.^176^ These gene expression signatures could be particularly important if—similar to melanoma—parameters such as YAP/TAZ expression or their nuclear localization turn out to serve as poor predictors of YAP/TAZ activity.^176^ (3) Do different types of CM display different dependencies on Hippo/YAP/TAZ signalling? To the best of our knowledge, a comprehensive investigation, using primary cells/cell lines and human tissue sample stratified according to the current WHO classification of human CM has not been performed. (4) How can we target YAP/TAZ‐dependencies in skin cancer? In addition to the studies that comprehensively investigate the cellular functions of YAP/TAZ and their transcriptional outputs, translational efforts will also benefit from focused proteomics studies aimed at characterizing the YAP/TAZ interactome in skin cancer cells. This could lead to the discovery of YAP/TAZ‐associated proteins that can possibly be targeted by existing compounds.

## AUTHOR CONTRIBUTIONS

GW and UJ conceptualized the manuscript. AH prepared figures and Tables. AH, JB, BF, SB, UJ and GW performed literature review and wrote different paragraphs.

## FUNDING INFORMATION

This work was supported by grants from the Biotechnology and Biological Sciences Research Council (grant BB/T012978/1), the Academy of Medical Sciences (grant SBF005\1005) and the British Skin Foundation (grant 036/S/18)(to Gernot Walko).

## CONFLICT OF INTEREST

The authors declare no conflict of interest.

## Data Availability

Data sharing is not applicable to this article as no new data were created or analyzed in this study.
